# The effect of a 5-year hand hygiene initiative based on the WHO multimodal hand hygiene improvement strategy: an interrupted time-series study

**DOI:** 10.1186/s13756-020-00732-7

**Published:** 2020-05-27

**Authors:** Yumi Suzuki, Motoko Morino, Ichizo Morita, Shigenori Yamamoto

**Affiliations:** 1 Department of Pediatrics, National Hospital Organization (NHO) Shimoshizu National Hospital, 934-5 Shikawatashi, Yotsukaido, 284-0003 Chiba Japan; 2 Division of Infection Control, NHO Shimoshizu National Hospital, Yotsukaidou, Japan; 3 Department of Nursing, NHO Shimoshizu National Hospital, Yotsukaidou, Japan; 4 Japanese Red Cross Toyota College of Nursing, 12-33 Nanamagari Hakusancho, Toyota, 471-8565 Aichi Japan

**Keywords:** Infection prevention, Hand hygiene, Alcohol-based hand rub, Compliance, Multimodal initiative, World Health Organization

## Abstract

**Background:**

A World Health Organization (WHO) guideline-based multimodal hand hygiene (HH) initiative was introduced hospital-wide to a nonteaching Japanese hospital for 5 years. The objective of this study was to assess the effect of this initiative in terms of changes in alcohol-based hand rub (ABHR) consumption and the Hand Hygiene Self-Assessment Framework (HHSAF) score.

**Methods:**

The consumption of monthly hospital-wide ABHR was calculated in L per 1000 patient days (PDs). The change in ABHR consumption was analysed by an interrupted time series analysis with a pre-implementation period of 36 months and an implementation period of 60 months. The correlation between annual ABHR consumption and the HHSAF score was estimated using Pearson’s correlation coefficients.

**Results:**

The annual ABHR consumption was 4.0 (L/1000 PDs) to 4.4 in the pre-implementation period and 10.4 to 34.4 in the implementation period. The HHSAF score was 117.5 (out of 500) in the pre-implementation period and 267.5 to 445 in the implementation period. A statistically significant increase in the monthly ABHR consumption (change in slope: + 0.479 L/1000 PDs, *p* <  0.01) was observed with the implementation of the initiative. Annual ABHR consumption was strongly correlated with the annual HHSAF score (r = 0.971, *p* <  0.01).

**Conclusions:**

A 5-year WHO-based HH initiative significantly increased ABHR consumption. Our study suggested that the HHSAF assessment can be a good process measure to improve HH in a single facility, as ABHR consumption increased with the HHSAF score.

## Background

Hand hygiene (HH) plays a key role in preventing hospital-acquired infections, as it prevents the spread of infectious organisms from patient to patient through the contamination of healthcare workers (HCWs)’ hands [1–3]. The World Health Organization (WHO) published a multimodal strategy to improve hand hygiene compliance in 2009 [4], and improved HH has been reported from several areas following national and subnational HH campaigns based on the WHO guidelines [3, 5–7]. However, there have been no active national or sub-national initiatives in Japan, and a study on HH compliance in 4 Japanese teaching hospitals reported an overall compliance of 19% [8]. Additionally, paediatric long-term care facilities have shown low HH compliance [9, 10]. Infection control in such settings is considered challenging because patients with heavy medical needs, such as ventilators and tube feeding, also require therapeutic and social activities, resulting in frequent interactions with many HCWs and nonmedical staff [11].

The WHO multimodal HH strategy includes 3 main concepts: the “My 5 Moments for Hand Hygiene” approach, the “five strategy components”, and the “step-wise approach” [12]. The “My 5 Moments for Hand Hygiene” approach highlights indications for moments for HH. The “five strategy components” refer to the implementation of multiple actions to tackle different obstacles and behavioural barriers to improving HH. These include Component 1: System Change, Component 2: Training and Education, Component 3: Evaluation and Feedback, Component 4: Reminders in the Workplace, Component 5: Institutional Safety Climate for Hand Hygiene. These five components stand for both the “five elements of the WHO multimodal Hand Hygiene Improvement Strategy” in the Guide to Implementation (GTI) [12], and the “five components of the Hand Hygiene Self-Assessment Framework (HHSAF) [13]”. The “step-wise approach” helps to develop and plan the hand hygiene improvement programme over time based on a rational sequence of activities. This approach includes the following five steps: Step 1: Facility preparedness, Step 2: Baseline evaluation, Step 3: Implementation, Step 4: Follow-up evaluation, and Step 5: Ongoing planning and review cycle. As the original concept of the WHO strategy is intended to make improvements in HH sustainable, not to be used as a single campaign, the entire cycle is recommended to be repeated for 5 years [12]. Some previous studies have reported the results from adopting some parts of this strategy, the majority of which focused only on the “My 5 Moments for Hand Hygiene” approach and the “five strategy components” [14], while several focused on the “step-wise approach” [15, 16]. In spite of the original recommendations in the guidelines, however, to date no studies have implemented both the “five strategy components” and the “step-wise approach” for 5 consecutive years.

The Hand Hygiene Self-Assessment Framework (HHSAF) [13] is a validated, systematic tool with which a situational analysis of hand hygiene promotion and practices in each health-care facility can be obtained [17]. The HHSAF acts as a diagnostic tool, identifying key issues requiring attention and improvement. Repeated use of the HHSAF also allows visualization of progress over time [13]. This tool has now been adopted in more than 90 countries [18]. We recorded our annual HHSAF scores for 5 years, which enabled us to visualize our progress over time and to analyse the correlation of the score with alcohol-based hand rub (ABHR) consumption, a surrogate marker for HH compliance.

We decided to introduce a HH initiative following the WHO HH strategy as a whole, including the “step-wise approach” and the HHSAF in our hospital, in which no systematic measures on HH had been implemented before. The aim of this study was to assess the effect of a hospital-wide WHO guideline-based HH initiative for 5 years. We also examined the correlation between the HHSAF score and ABHR consumption as an exploratory analysis.

## Methods

### Study design and statistical methods

An interrupted time series analysis was performed to examine the change in monthly ABHR consumption with the implementation of the HH initiative. The correlation between the HHSAF score and ABHR consumption was assessed using Pearson’s correlation coefficients. The analyses were performed using IBM SPSS version 24 (IBM; Armonk, New York, USA).

### Data collection

The HH initiative started in April 2014 and continued for 5 years until March 2019. The pre-implementation period was from April 2011 to March 2014 (36 months), and the implementation period was from April 2014 to March 2019 (60 months). Monthly and annual ABHR consumption (L per 1000 patient days; L/1000 PDs) was calculated by dividing the total amount of ABHR delivered from the pharmacy, hospital-wide, by the corresponding periods’ patient census (patient hospitalization days). Monthly ABHR consumption and patient hospitalization days are shown in the Supplementary file. ABHR consumption data were collected from April 2014 to March 2019 each month in a prospective manner, and data from April 2011 to March 2014 were collected retrospectively.

The annual HHSAF score was calculated at the end of each fiscal year from 2013 to 2019. Scores for 2011 and 2012 were calculated retrospectively. The same infection control nurse (ICN) and infection control doctor (ICD), both in charge of the hospital-wide HH initiative, confirmed the scores together throughout the study period.

### Setting

The study was conducted in the 440-bed NHO Shimoshizu National Hospital, located in Chiba, Japan. This hospital is not a typical ‘teaching hospital’, except for some specialties that have limited teaching functions. Four wards, with a total of 200 beds, are mainly for secondary care (mean length of stay: 9.4 to 20.0 days). The proportion of private rooms is between 42.9 and 66.6%. Five wards, with a total of 240 beds, provide long-term care mainly for patients with complex chronic medical conditions and require heavy medical care, such as mechanical ventilation and/or tube feeding (mean length of stay: 99.5 to 898.6 days). These wards are similar to paediatric long-term care facilities referenced in [9, 10]. The proportion of private rooms is between 30.0 and 42.9%. On average, over a hundred patients a day required ventilators (including non-invasive positive pressure ventilators used only overnight) during the study period, although there are no intensive care unit (ICU) beds in the hospital. Patients in the long-term care wards have direct contact with several recreational staff members every day, in addition to clinical staff for medical care. The link nurses (LNs: young nurses in charge of infection control within their ward) calculated their ideal HH events per PD for each ward based on a survey established by the ICN, referring to a previous study that used a nursing activity recording system [19]. Every year, all nurses in each ward recorded their nursing activities for 24 consecutive hours for more than 3 separate days. The LNs calculated the total number of HH opportunities required per PD by analysing the nursing workload data with the “5 Moments” indications and then dividing this by the number of patients hospitalized. The average data for the 3 days, which was considered to be the usual workload for the ward, was used as the estimated ideal HH events per PD. The ideal number of HH events per PD estimated for each ward ranged from 15 to 25 in the acute care wards and from 40 to 60 in the long-term wards. The ideal hospital-wide HH events per PD was approximately 38, obtained by taking the average of the values for all wards. As 1.3 ml of ABHR is dispensed in each HH event, adequate hospital-wide ABHR consumption for our goal of 100% compliance was estimated to be approximately 50 L/1000 PDs. The amount of 1.3 ml was determined by the product manufacturer through their own experiments [20, 21]. At least one bottle of ABHR was placed at the entrance of each patient’s room, each of which had up to a maximum of 4 beds, throughout the study period. In the pre-implementation period, some wards had posters with phrases such as “One Procedure, One Hand Wash”, which had not been changed for years. The LNs checked hand washing procedures in training sessions that were held twice a year for each ward by rubbing a solution on the hands that glows under UV light. These sessions were the only regular HH training sessions conducted in the hospital. HH compliance was monitored by direct observation irregularly once to a few times a year, but the auditors had received only very limited training prior to the implementation of the initiative. No systematic HH initiatives had been implemented, and nobody had overviewed the whole HH improvement programme of the hospital before the study. The hospital has an infection control team (ICT) with 0.3 full-time equivalent (FTE) ICD throughout the study and 1.0 FTE ICN from January 2013 to the end of the study. All HCWs who have direct contact with the patients were included as participants of the study.

### Interventions

We conducted an original 5-year hospital-wide HH initiative from April 2014 to March 2019, implementing activities from all five strategy components, with no incentives given to the participants. Our programme was based on the Guide to Implementation (GTI) [12], but some minor changes and local adaptations were made to match our hospital culture, as was recommended. In addition to translating the English tools, we prepared original posters and reminders with pictures hand drawn by the ICT and educational videos with the hospital staff as the actors, filmed in the actual hospital setting to enhance comprehension. The details of the activities implemented each year are shown in Table 1.

**Table 1:** Details of the activities corresponding to the five strategy components implemented each year

	Activities of the initiative	Year 1	Year 2	Year 3	Year 4	Year 5
Component 1	Distribution of personal shoulder bags for ABHR bottles	×	×	×	×	×
	Automatic ABHR dispensers at the door of each room		×	×	×	×
	Forms of ABHR (gel, foam, aroma, etc.)		×	×	×	×
	Hand moisturizer				×	×
Component 2	HH leader training sessions	×				
	WHO HH guidelines and tool kits in all computers	×	×	×	×	×
	Regular HH training sessions for all staff	×	×	×		
	Mandatory HH training sessions for all staff				×	×
	Original HH training video made by the ICT & ICMs		×	×	×	×
	E-learning using the original training video			×	×	×
	On-the-job direct observation training for ICMs & LNs			×	×	×
	Hands on training sessions					×
Component 3	ABHR consumption monitoring by the ICT (monthly)	×	×	×	×	×
	ABHR consumption monitoring by LNs of each ward		×	×	×	×
	Personal ABHR consumption monitoring by LNs				×	×
	HH events per day survey by LNs	×	×	×	×	×
	Direct observation by the ICT (twice a year per ward)	×	×	×	×	×
	Direct observation by LNs			×	×	×
	Perception survey for senior executive managers	×				
	Perception survey for all staff	×			×	
	Knowledge survey for all staff	×		×		
Component 4	5 Moments reminders on ABHR bottles	×	×	×	×	×
	HH procedure posters	×	×	×	×	×
	Posters for each HH campaign		×	×	×	×
	5 Moments posters with hand drawn pictures			×	×	×
	Hand rub procedure reminders with hand drawn pictures					×
	Reminders made by LNs for each ward					×
Component 5	Letter to the director	×				
	Letters to the head of each department and ward	×				
	Assignment of ICMs as HH champions	×	×	×	×	×
	Selection of “HH masters” as role models			×	×	×
	HH campaign twice a year (May & October)	×	×	×	×	×
	Campaign poster with a picture of the director			×	×	×
	Campaign poster with a picture of the staff members				×	×
	Institutional target	×	×	×	×	×
	Presentation sessions to share activities on HH			×	×	×
	Newsletters with issues on HH			×	×	×
	Inclusion of HH as part of the buddy training system					×

*ABHR* alcohol-based hand rub, *Component 1* System Change, *Component 2* Training and Education, *Component 3* Evaluation and Feedback, *Component 4* Reminders in the Workplace, *Component 5* Institutional Safety Climate for Hand Hygiene, *HH* hand hygiene, *ICT* infection control team, *ICM* infection control manager, *LN* link nurse

We repeated the five steps of the step-wise approach each fiscal year. In the GTI, the length of time recommended for each step is as follows: Step 1, 2 months; Steps 2 and 3, 3 months; and Steps 4 and 5, 2 months. In our original programme, we minimized the length of time for Steps 2 and 4 by applying the data from the previous year as the baseline and the annual data as the follow-up data. The outline of each step, determined according to the GTI, is summarized below; 201X stands for 2014 to 2018, and 201X + 1 stands for 2015 to 2019.

Step 1 (April to May 201X): Facility preparedness: The infection control committee (ICC: the highest decision-making body on infection control) decided upon and declared the hospital-wide implementation of the WHO HH strategy, the annual aim, and the hospital-wide target amount of ABHR consumption. The ICT was assigned as the main promotion team, and the infection control managers (ICMs: the field leaders on infection control) were assigned to work as the HH leaders (champions) for each department and ward.

Step 2 (May 201X): Baseline evaluation: The hospital-wide ABHR consumption from the previous year was applied as the baseline. At least one direct observation session for each ward was completed by the ICT within the month.

Step 3 (May 201X to March 201X + 1): Implementation: Tools and activities from all five strategy components were selected and adapted as described in Table 1. Hospital-wide activities were mainly led by the ICT, and local activities were mainly led by the ICMs. LNs worked with the ICMs in the nursing departments.

Step 4 (March to April 201X + 1): Follow-up evaluation: The ICT reported the annual (April 201X to March 201X + 1) ABHR consumption, the findings from the direct observations, and the annual HHSAF score.

Step 5 (March to April 201X + 1): Ongoing planning and review cycle: The achievement of the year and the remaining challenges were evaluated by the ICC. The institutional aim, the target amount, and the target activities for the next year were planned by the ICT for continuous improvement.

## Results

### Participants

A total of 402 HCWs worked full time in the hospital at the final month of the study and were all included as participants. This group comprised 37 physicians, 261 members of the nursing staff, 20 rehabilitation therapists, and 20 members of the recreational staff. The number of workers varied within the study period but showed no increasing or decreasing tendencies. The average number of monthly patient hospitalization days throughout the study period was 9970. The average was 10,482 for the pre-implementation period and 9662.9 for the implementation period.

### Implementation of the initiative

Table 2 shows the details of the five steps of the step-wise approach each year. For the first 2 years, most activities were planned and conducted directly by the ICT. In the following years, plans were made so that the ownership of the HH initiatives would be transferred to the ICMs, the front-line champions, to let them lead local activities.

**Table 2:** Details of the five steps of the step-wise approach in the 5-year cycle

Year 1 (April 2014 to March 2015)	
Steps 1 & 2	•Annual aim: develop an effective system and provide adequate ABHR to each point of care. •Annual target amount: 10 L/1000 PDs, approximately double the amount of the previous year.
Step 3	•HH initiatives were planned and executed mainly by the ICT.
Steps 4 & 5	•Moment 1 (before touching the patient) was found to be the most missed throughout the hospital. •Target amount was achieved. •Participation of the field HH leaders such as ICMs and the LNs remained a challenge.
Year 2 (April 2015 to March 2016)	
Steps 1 & 2	•Annual aim: Improve compliance for Moment 1. •Annual target amount: 15 L/1000 PDs, referring to the report by Pittet et al. [1]
Step 3	•Initiatives were still mainly planned and executed by the ICT, but ICMs and LNs were encouraged to take a more active role, especially in the Components 2 and 3.
Steps 4 & 5	•Compliance differences between individuals became apparent. •Target amount was achieved. •The need for different approaches to match the differences in the individuals’ abilities was recognized, such as defining role models and providing adequate support to individuals having difficulties.
Year 3 (April 2016 to March 2017)	
Steps 1 & 2	•Annual aim: Encourage individual support for staff with low compliance and promote the activities of the staff with high compliance, at each local field level. •Annual target amount: 25 L/1000 PDs, 1/2 the estimated adequate ABHR consumption.
Step 3	•Many tools from Component 5 were utilized to reinforce field-based initiatives.
Steps 4 & 5	•The compliance differences between the wards and departments became apparent. •Target amount was 91.6% achieved. •Field-level HH initiatives of fields with high compliances should be shared.
Year 4 (April 2017 to March 2018)	
Steps 1 & 2	•Annual aim: Share effective initiatives between wards and departments, focusing on Moment 1 again. This moment was selected as it was a common moment for every HCW, and sharing was expected to be effective. •Annual target amount: 30 L/1000 PDs, 3/5 the estimated adequate ABHR consumption.
Step 3	•Effective activities were shared in ICM meetings. The ICT provided 4 weeks of intensive support to several wards experiencing difficulties.
Steps 4 & 5	•HH was found to be missed in certain routine procedures, which differed between fields. •Target amount was 99% achieved. •Voluntary activities of the ICMs and LNs should be further encouraged.
Year 5 (April 2018 to March 2019)	
Steps 1 & 2	•Annual aim: Focus on HH in the routine work of each ward and department. •Annual target amount: 33 L/1000 PDs, 2/3 the estimated adequate ABHR consumption.
Step 3	•ICMs and LNs reviewed and focused on the HH moment that tended to be missed in their everyday routine work procedures.
Steps 4 & 5	•Target amount was achieved. •HHSAF assessment showed that Component 5 had the most room for improvement.

*ABHR* alcohol-based hand rub, *ICC* infection control committee, *ICM* infection control manager, *ICT* infection control team, *HCW* health care worker, *HH* hand hygiene, *HHSAF* Hand Hygiene Self-Assessment Framework, *LN* link nurse, *PD* patient day, *Step 1* Facility preparedness, *Step 2* Baseline evaluation, *Step 3* Implementation, *Step 4* Follow-up evaluation, *Step 5* Ongoing planning and review cycle

Table 3 shows the annual ABHR consumption and HHSAF score. The annual ABHR consumption ranged from 4.0 (L/1000 PDs) to 4.4 in the pre-implementation period and from 10.4 to 34.4 in the implementation period. The HHSAF score was 117.5 (out of 500) in the pre-implementation period and 267.5 to 445 in the implementation period. The HHSAF included another component; the “Leadership Criteria” for facilities that had reached the advanced level (score 376–500) of HH, but this was not assessed during this study period.

**Table 3:** Annual ABHR consumption and HHSAF score in each year

	Intervention Year	Annual ABHR Consumption (L/1000 PDs)	HHSAF Score (/500)
Pre-implementation	Year −3 (Apr-11 to Mar-12)	4.4	117.5
	Year −2 (Apr-12 to Mar-13)	4.0	117.5
	Year −1 (Apr-13 to Mar-14)	4.2	117.5
Implementation	Year 1 (Apr-14 to Mar-15)	10.4	267.5
	Year 2 (Apr-15 to Mar-16)	17.7	310.0
	Year 3 (Apr-16 to Mar-17)	22.9	380.0
	Year 4 (Apr-17 to Mar-18)	29.6	410.0
	Year 5 (Apr-11 to Mar-19)	34.4	445.0

*ABHR* alcohol-based hand rub, *HHSAF* Hand Hygiene Self-Assessment Framework, *PD* patient day

Table 4 shows the details of the HHSAF score.

**Table 4:** Details of the Hand Hygiene Self-Assessment Framework Score

		Year −3	Year − 2	Year − 1	Year 1	Year 2	Year 3	Year 4	Year 5
Component 1	1.1	10	10	10	30	50	50	50	50
	1.2	5	5	5	5	5	5	5	5
	1.3	10	10	10	10	10	10	10	10
	1.4	10	10	10	10	10	10	10	10
	1.5	10	10	10	10	10	10	10	10
	1.6	10	10	10	10	10	10	10	10
	add	0	0	0	0	0	0	0	0
	subtotal	55	55	55	75	95	95	95	95
Component 2	2.1a	10	10	10	10	10	10	20	20
	2.1b	20	20	20	20	20	20	20	20
	2.2a	0	0	0	5	5	5	5	5
	2.2b	0	0	0	5	5	5	5	5
	2.2c	0	0	0	5	5	5	5	5
	2.2d	0	0	0	5	5	5	5	5
	2.3	0	0	0	0	0	15	15	15
	2.4	0	0	0	0	0	0	0	15
	2.5	0	0	0	0	0	0	10	10
	subtotal	30	30	30	50	50	65	85	100
Component 3	3.1	0	0	0	0	0	0	10	10
	3.2a	0	0	0	0	0	5	5	0
	3.2b	5	5	5	5	5	5	0	5
	3.3a	0	0	0	5	5	5	5	5
	3.3b	0	0	0	0	0	0	0	0
	3.3c	0	0	0	0	0	5	5	5
	3.4a	5	5	5	10	10	10	10	10
	3.4b	0	0	0	15	20	20	20	20
	3.5a	0	0	0	5	5	5	5	5
	3.5bi	0	0	0	0	7.5	7.5	7.5	7.5
	3.5bii	0	0	0	0	7.5	7.5	7.5	7.5
	subtotal	10	10	10	40	60	70	75	75
Component 4	4.1a	0	0	0	20	20	20	25	25
	4.1b	5	5	5	5	5	5	5	15
	4.1c	7.5	7.5	7.5	7.5	10	10	10	10
	4.2	0	0	0	0	0	10	10	10
	4.3	0	0	0	10	10	10	10	10
	4.4	0	0	0	0	0	10	10	10
	4.5	0	0	0	15	15	15	15	15
	subtotal	12.5	12.5	12.5	57.5	60	80	85	95
Component 5	5.1a	5	5	5	5	5	5	5	5
	5.1b	5	5	5	5	5	5	5	5
	5.1c	0	0	0	0	0	5	5	5
	5.2a	0	0	0	10	10	10	10	10
	5.2b	0	0	0	0	0	0	0	0
	5.2c	0	0	0	5	5	5	5	5
	5.3	0	0	0	10	10	10	10	10
	5.4a	0	0	0	5	5	5	5	5
	5.4b	0	0	0	0	0	5	5	5
	5.5a	0	0	0	0	0	0	0	0
	5.5b	0	0	0	0	0	0	0	0
	5.6a	0	0	0	0	0	5	5	5
	5.6b	0	0	0	5	5	5	5	5
	5.6c	0	0	0	0	0	5	5	5
	5.6d	0	0	0	0	0	5	5	5
	5.6e	0	0	0	0	0	0	0	5
	5.6f	0	0	0	0	0	0	0	5
	subtotal	10	10	10	45	45	70	70	80
total HHSAF score		117.5	117.5	117.5	267.5	310	380	410	445

*Component 1* System Change, *Component 2* Training and Education, *Component 3* Evaluation and Feedback, *Component 4* Reminders in the Workplace, *Component 5* Institutional Safety Climate for Hand Hygiene, *HHSAF* Hand Hygiene Self-Assessment Framework

### Monthly ABHR consumption

**Fig. 1:**
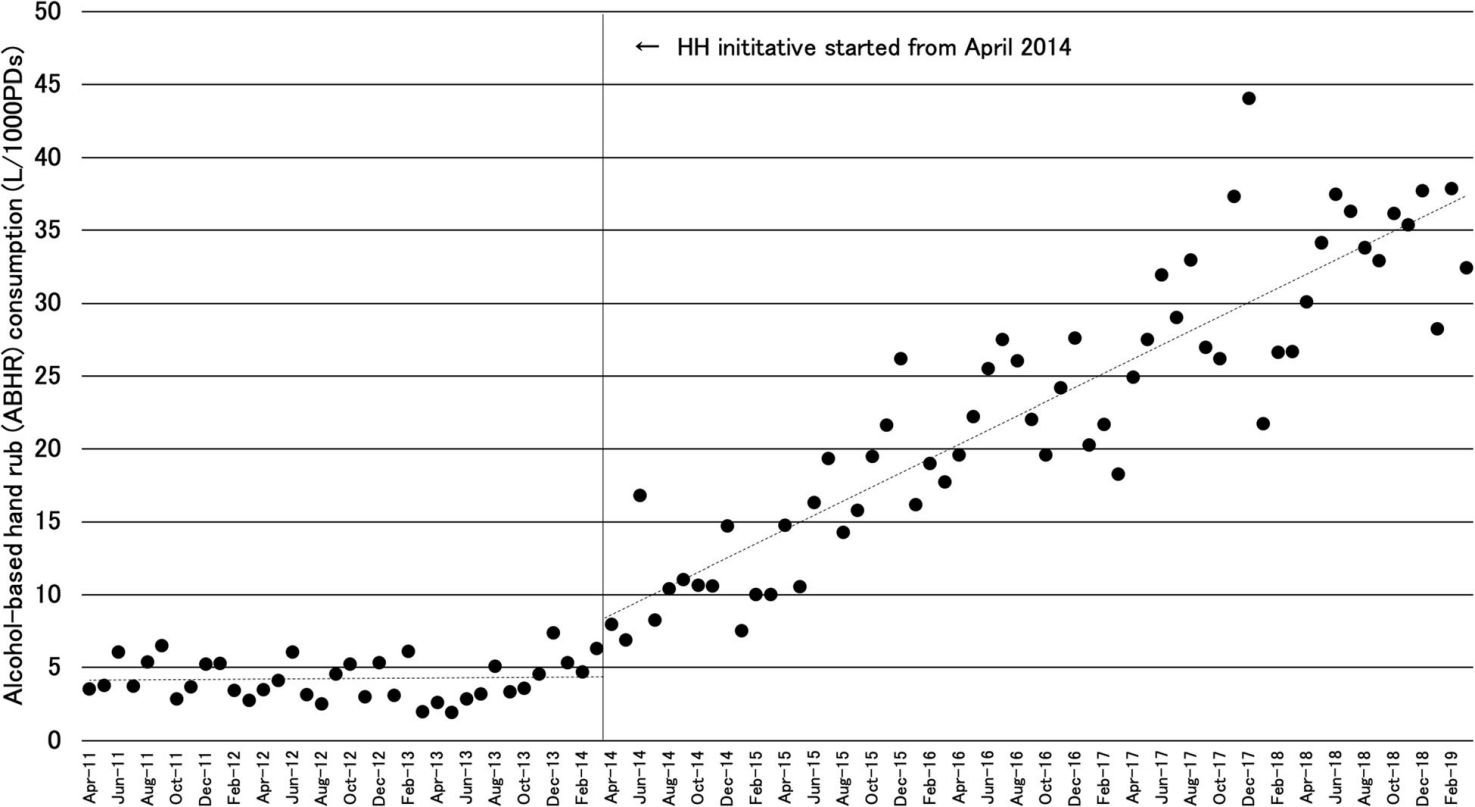
Trends change in ABHR consumption before and after the implementation of the HH initiative. *ABHR* alcohol-based hand rub, *HH* hand hygiene, *PD* patient day

Figure 1 shows the trend of monthly ABHR consumption. The r value for the regression model was 0.958, and the adjusted r^2^ value was 0.916. The baseline ABHR consumption in the pre-implementation period was estimated to be 4.3 L/1000 PDs. This was stable with no observable trend in the baseline segment. At the start of the implementation of the hospital-wide HH initiative in April 2014, an immediate increase of 4.4 L/1000 PDs, approximately doubling the baseline ABHR consumption, was observed. Furthermore, there was a significant slope change of + 0.479 (95% confidence interval: 0.359 to 0.599, *p* <  0.001) L/1000 PDs in the ABHR consumption before and after the implementation of the initiative. Table 5 summarizes the estimates from the segmented regression model.

**Table 5:** Parameter estimates, 95% CIs and *P*-values from segmented regression model describing the trends of monthly ABHR consumption

	Coefficient	95% CI	*P*-value^a^
Intercept	4.344	2.032–6.656	< 0.001
Baseline trend	0.006	−0.103-0.115	0.919
Level change from last point in the pre-implementation to the first point in the implementation phase	4.387	1.499–7.276	0.003
Slope change from pre-implementation to implementation	0.479	0.359–0.599	< 0.001

*ABHR* alcohol-based hand rub, *CI* confidence interval

The model describes trends and change in the error rate during the pre-implementation, and implementation phases

^a^Calculated using Student’s t-test

### Annual ABHR consumption and HHSAF scores

**Fig. 2:**
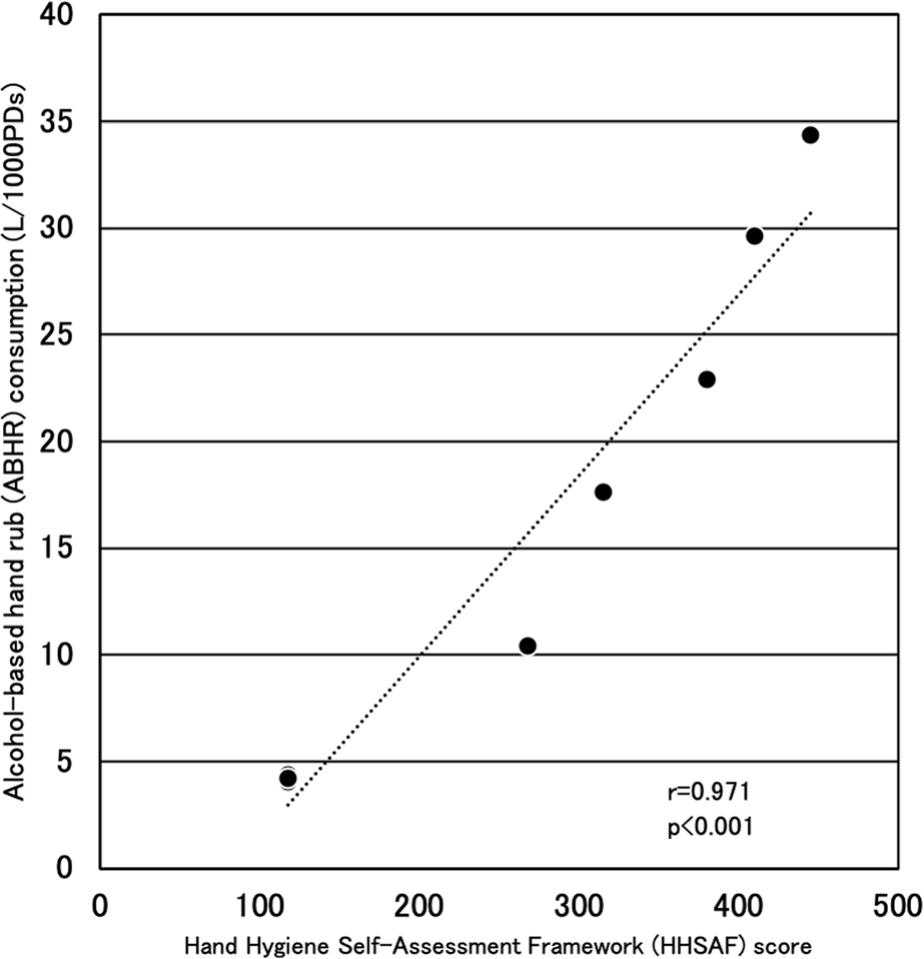
Relationship between annual ABHR consumption and HHSAF score. *ABHR* alcohol-based hand rub, *HHSAF* Hand Hygiene Self-Assessment Framework, *PD* patient day

Table 3 shows the annual ABHR consumption and HHSAF score. Figure 2 shows that a significant positive correlation (r = 0.971, *p* < 0.001) was found between the annual ABHR consumption and HHSAF score.

## Discussion

A 5-year WHO-based HH initiative significantly increased ABHR consumption in a nonteaching secondary hospital in Japan with long-term care wards. In addition, HHSAF scores were also increased with a significant positive correlation with ABHR consumption.

The WHO guidelines recommend repeating the entire cycle of the step-wise approach for a minimum of 5 years. Despite the recommendation, the majority of previous studies reporting the effect of a multimodal WHO-based HH programme focused only on the five strategy components. Although several studies followed the step-wise programme [15, 16] or a 5-year programme [22, 23], to the best of our knowledge, there have been no previous studies reporting a 5-year programme with five cycles of the step-wise approach. Our 5-year initiative resulted in an eight-fold increase in ABHR consumption, from 4.2 to 34.4 L/1000 PDs, which is similar to the increase reported by Pittet, from 4.1 to 30.6 L/1000 PDs, over 7 years [24]. Additionally, our study is the first to report the 5-year change in the HHSAF score in a single facility, which showed a nearly four-fold increase, from 117.5 to 445 points.

The rationale for the importance of a 5-year programme is not given in the WHO guidelines. The target amount of ABHR consumption recommended in the guidelines is 20 L/1000 PDs (HHSAF: 3.3c), and in Pittet’s study, published prior to the publication of the guidelines, a period of 5 years was necessary to increase ABHR consumption from 4.1 to 20.8 L/1000 PDs [24]. Additionally, previous studies have suggested that behavioural changes are important in the improvement of HH [25–27] and that this improvement took time, sometimes years. Similar results were reported from a 6-year initiative in a tertiary teaching hospital [28] and a 4-year initiative in a paediatric long-term care facility [29].

In our initiative, we put effort into increasing local field staff involvement each year, as our long-term patients had frequent daily contact with staff members from many different departments, and the effective measures differed between them. During the first 3 years, the ICT began implementing new systems for the ICMs to work as active field HH leaders. Then, in the last 2 years, the ICMs led the “local, focused implementation” of the HH initiative with the support of the LNs. From these experiences, we retrospectively recognized that 5 years was a reasonable length of time to change systems and to embed frontline staff engagement for local focused implementation.

Our annual hospital-wide HH compliance, monitored by direct observation, nearly tripled in the first 2 years after initiation of the intervention, which then ranged from 68 to 70% from the second year to the fifth year. We continuously conducted direct observations, but we did not include any of these data as an outcome or quantitative measure, as we were unable to regard our quantitative data as reliable for several reasons. First, prior to the implementation of the initiative, there was a lack of trained auditors who could conduct the direct observations accurately. Second, because our initiative was carried out with no additional staff reinforcement or high-tech recording devices, only overt observations were possible. We observed the “Hawthorn effect” in most of the direct observations, which became more obvious in the latter phase of the initiative. In the final years of the intervention, the behaviours of the HCWs began to change within minutes of the start of each observation session. Third, there was also a large ‘observer bias’, as the auditors tended to follow HCWs with low compliances to determine the main obstacles for improving HH compliance.

On the other hand, we were able to obtain much informative qualitative data by “examining in detail the barriers and opportunities to increase HH compliance” [30]. Therefore, rather than emphasizing the quantitative data, we focused on showing where improvement was most required specifically for the field staff, preparing effective training programmes for the following year, and determining our annual institutional aim based on the findings from the direct observations. For example, the annual aim chosen for the second year was to “Improve compliance for Moment 1 (before touching the patient)”, as this was found to be the most missed of the “5 Moments” throughout the hospital during the previous year.

The current gold standard for HH compliance monitoring is unobtrusive direct observation [31]. However, conducting unobtrusive observations continuously and daily for years is difficult for most hospitals and cannot be widely recommended in terms of feasibility. High-tech recording devices are available, but most nonteaching hospitals in Japan cannot afford them. It is also said that direct observations can only catch a very small proportion of the actual HH events performed [32]. ABHR consumption, a surrogate marker for monitoring HH compliance, can be monitored easily and continuously for years and can also give a 24-h picture of compliance for all clinicians [33]. ABHR consumption monitoring has been officially recommended in “The surveillance procedures for small and medium sized medical facilities” since 2009 in Japan and has also been applied by the European Centre for Disease Prevention and Control [34] for standardized surveillance purposes. Many reports from Europe [35–38] and a report from Africa [39] have indicated the adoption of indirect monitoring of HH activity based on an ABHR consumption system. As long as direct observations are also conducted for qualitative measures and no punitive approaches are taken, ABHR consumption monitoring may be considered as a practical measure, especially for assessing improvement in long-term initiatives, for facilities with limited resources.

The WHO guidelines state that Step 5 of the stepwise approach is a crucial step for developing long-term plans to ensure that improvement is sustained and progresses. Reviewing our present position with ABHR consumption, findings from the direct observations, and the HHSAF score each year provided us with a bird’s eye view of what we have accomplished and what is left to be done. The HHSAF helps to identify key issues requiring attention and the resources and tools useful for achieving them [13, 17]. We referred to the “Template Action Plan” (TAP) [40] that was prepared for our HH level when starting our initiative. However, once our HH initiative had started, we made our annual plans for the following year by choosing tools and activities mainly for the components that scored lowest in the HHSAF. This was effective not only because appropriate activities that were required at the moment were selected but also because it provided the frontline staff members convincing reasons why this particular activity was chosen for the year. By continuing this process, we were able to achieve high HHSAF scores for all five strategy components, which further increased the capacity for improvement. As we have reached the Advanced HH level, we are now working on the ‘Leadership Criteria’ of the HHSAF.

We found that ABHR consumption and HHSAF score were significantly positively correlated. We expected that both would increase as a result of the initiative but did not expect that the 2 variables would show such a strong correlation. A prior study from Japan [41] suggested that compliance would be improved by increasing the HHSAF score. In this study, the HH compliance rate obtained by direct observation and the HHSAF scores were compared between 3 Japanese hospitals; they ranked in the same order for both measurements. Our findings supported their results and indicated the possibility of adopting the HHSAF as a process measure in a single facility. This may be useful for some other hospitals as well, especially for those with low baseline compliance and HHSAF scores, when conducting a long-term initiative. Further reports from other hospitals and multi-centre reports are needed to confirm this.

Our study was challenging in several aspects. Although national and subnational HH initiatives based on the WHO HH strategy have been introduced in many countries [3, 5–7], such full-scale initiatives had not been introduced in Japan by the time of this study, and the HH initiatives were left to each hospital’s own efforts. In addition, having long-term care wards with many patients on ventilators, as well as daily recreational activities, made our situation even more complex. Furthermore, similar to many other nonteaching hospitals in our country, we could not afford additional personnel for covert observation or high-tech recording devices to assess HH compliance. However, by tracking the ABHR consumption, together with the HHSAF score as a process measure, we were able to complete our HH initiative successfully. Along with working on all five strategy components, repeating the review process at Step 5 of the step-wise approach for 5 consecutive years may have been one of the most important elements of our initiative. This has become a sustainable routine for us over this period of time, and we will continue repeating the cycles of the step-wise approach to sustain our improvement in HH practice.

There are several limitations in this study. First, this is a report from a single Japanese hospital, which provides long-term care for many patients with heavy medical needs and with no previous effective HH campaigns or initiatives. The amount of ABHR required in our hospital may be greater than that in many other hospitals that do not need to set such a high target. In addition, hospitals with higher HH compliance at baseline may not experience such an increase in ABHR consumption.

Second, we could not continuously record direct HH compliance. Some amount of ABHR may have been discarded or used incorrectly. As we did not provide incentives or punishment for the amount of ABHR consumed, we assume that there was not much advantage for each staff member to discard the substance. Although we found from our direct observations that the staff members with high ABHR consumption tended to use ABHR adequately, the possibility of discarded substance and incorrect use cannot be ruled out. In addition, the amount of ABHR that was used by patients and visitors was included in the ABHR consumption. As patient involvement in hand hygiene is recommended to improve the culture and climate of HH and to reduce hospital-acquired infections, we included patient/visitor ABHR consumption as part of the total HH improvement in our hospital.

Third, the amount we adopted as the adequate amount per HH event, 1.3 ml, is much less than the 3 ml said to be recommended by most ABHR manufacturers, and even larger amounts were recommended for HCWs with large hands in a study from Europe [42]. The WHO guidelines recommend 20 to 30 s for each hand rub event, but some recent reports show that a 15-s application time is equal to a 30-s application in terms of wettability of hands [43] and is not inferior in terms of reducing bacterial counts on hands under experimental conditions [44]. Shortening the duration of ABHR application to 15 s may also improve compliance [45]. In our study, the 1.3 ml ABHR that we used stayed wet for 20 s during the routine rubbing procedure for most participants, which may be because of the types of formula we used (mainly gel type, with moisturizing ingredients) [20, 21] and/or the fact that Japanese HCWs tend to have smaller hands than European HCWs. For HCWs with larger hands whose hands do not stay wet for at least 15 s, we recommended 2 pushes (2.6 ml) per HH event, but this proportion and amount was not analysed in this study.

Fourth, the outbreaks that we experienced within this study period—a two-drug–resistant *Acinetobacter baumanii* outbreak in 2014 and a multiple-drug–resistant *Pseudomonas aeruginosa* (MDRP) outbreak in 2016—may have affected our results. Such outbreaks themselves can induce an increase in ABHR consumption, and the possibility of their influence cannot be excluded. However, the effects from these situations were expected to be temporary and limited to the ward in which the outbreak occurred. Our hospital-wide ABHR consumption continued to increase, regardless of the convergence of these outbreaks.

Fifth, the number of patient hospitalization days decreased between the pre-implementation period and the implementation period. This may be due to a change in the hospital policy in April 2014, which required a referral letter from every first visit patient. It is known that poor HH adherence is associated with higher patient-to-staff ratios [46], so the decrease in the number of patients may have had some influence on the increasing ABHR consumption per patient day. However, the 7.8% decrease in the mean number of patients alone could not have caused the eight-fold increase in the mean annual ABHR consumption (from 2013 to 2018), although it may have provided some positive effect.

Sixth, the HHSAF includes the amount of ABHR consumption as one of its scores. The maximum score given to the ABHR consumption is 5 points, which is 1% of the total score. HH compliance by direct observation is also included, with a maximum score of 30 points. Our score for direct HH compliance remained 20 points for the final 4 years. Altogether, our highest score for direct and indirect HH compliance was 25 points, which is 5% of the total score. This is not a large proportion; nevertheless, it cannot be said that the HHSAF score and ABHR consumption are completely independent variables.

## Conclusions

We successfully implemented a WHO-based multimodal HH initiative in a nonteaching, secondary and long-term care hospital in Japan. Working on all five strategy components, repeating the cycle of the five steps of the step-wise approach for 5 years and assessing the HHSAF score at Steps 4 & 5 each year resulted in a continuous increase in ABHR consumption. Our results suggested that the HHSAF score may well be considered for adoption as a process measure within a single facility, although further investigation is necessary.

## Supplementary information

**Additional file 1.** Monthly alcohol-based hand rub consumption and patient hospitalization days.

## Data Availability

All the data supporting the conclusions are available in the manuscript and in the supplementary file.

## Abbreviations

ABHR: Alcohol-based hand rub
FTE: Full time equivalent
GTI: Guide to Implementation
HCW: Health care worker
HH: Hand hygiene
HHSAF: Hand Hygiene Self-Assessment Framework
ICC: Infection control committee
ICD: Infection control doctor
ICM: Infection control manager
ICN: Infection control nurse
ICT: Infection control team
ICU: Intensive care unit
LN: Link nurse
MDRP: Multiple-drug–resistant *Pseudomonas aeruginosa*
NHO: National Hospital Organization
PD: Patient day
TAP: Template Action Plan
WHO: World Health Organization
